# Consolidation of Medical Schools

**Published:** 1857-09

**Authors:** 


*Consolidation of Medical Schools.*—The Miami Medical College and Medical College of Ohio, have been consolidated under the name &c. of the latter. Professors Judkins, Comegys, Foote and Mendenhall, of the Miami, go to the Ohio School; Professors Armor, Marshall, Varder and Foote, of the same, retiring.

